# Eribulin-induced resolution of high-risk basal cell carcinoma: implications for microtubule-targeting agents in BCC management: a case report

**DOI:** 10.2340/1651-226X.2026.44993

**Published:** 2026-02-03

**Authors:** Jan Lapins, Niki Radros, Karina Schultz, Ismini Vassilaki, Christina Linder Stragliotto, Hildur Helgadottir

**Affiliations:** a Department of Dermatology, Karolinska University Hospital, Stockholm, Sweden; b Dermatology and Venereology Unit, Department of Medicine, Solna, Karolinska Institutet, Stockholm, Sweden; c Theme Cancer, Karolinska University Hospital, Stockholm, Sweden; d Department of Oncology and Pathology, Karolinska Institutet, Stockholm, Sweden; e Department of Clinical Pathology and Cancer Diagnostics, Karolinska University Hospital, Stockholm, Sweden; f Department of Molecular Medicine and Surgery, Karolinska Institutet, Stockholm, Sweden

**Keywords:** Off-target antineoplastic effect, therapeutic repurposing, microtubule inhibition, tirbanibulin

## Introduction

Basal cell carcinoma (BCC) is a locally infiltrative skin cancer with high-risk behaviour when located near functionally critical structures, such as the eyes, nose, lips, and ears. Early detection allows for simple treatment, whereas delayed diagnosis increases the risk of deep infiltrative growth into the cartilage or bone, making treatment more difficult and challenging [1]. Standard treatment includes surgical excision with wide margins or Mohs micrographic surgery (MMS). When surgery is not feasible or fails to achieve clear margins, radiation therapy (external, interstitial, or contact brachytherapy), hedgehog inhibitors, or checkpoint inhibitors (CIs) are considered. Traditional chemotherapies, such as cisplatin, have shown limited efficacy [2]. While many patients are cured, a subset presents with recurrent or treatment-resistant disease, often requiring long-term management, resulting in significant functional and cosmetic effects. In locally advanced BCC, in which surgery and radiotherapy have already been attempted without success and are no longer feasible, the prospects for durable control are limited. Hedgehog pathway inhibitors (HHIs) induce tumour shrinkage in approximately half of patients, with higher response rates reported in some real-world cohorts, although complete and lasting responses are uncommon [3, 4]. CIs, such as cemiplimab, offer additional benefits after HHI failure but achieve meaningful and durable control in only a minority of patients [8]. Overall, a cure without further surgery or radiotherapy is rare. Therefore, new therapies targeting locally advanced or inoperable BCC are urgently required. We report a case of rapid and complete regression of a large, histologically confirmed infiltrative BCC on the nasal tip during systemic eribulin treatment for metastatic abdominal leiomyosarcoma.

## Case presentation

An 80-year-old woman with cardiovascular comorbidities, including atrial fibrillation (status post-His-ablation with pacemaker), hypertension, hyperlipidaemia, and previous transitory ichemic attack (TIA), with good performance status and quality of life, was referred for evaluation of a nasal tip lesion.

The patient with Fitzpatrick skin type II had multiple BCCs treated on her face, scalp, and trunk. In 2004, after unsuccessful surgical removal of a BCC on her right cheek and nasal dorsum, she received pulsed dose rate (PDR) contact brachytherapy with no recurrence in treated areas.

### BCC of the nasal tip

In 2012, a nasal tip lesion was treated with cryotherapy, and a biopsy in 2014 due to suspected recurrence showed actinic keratosis. In 2018, the patient underwent surgical excision of a BCC on the nasal tip with clear margins. In recent years, she had developed recurrent sores on the nasal bridge and tip, which were treated with topical fluorouracil in 2022. Despite this, symptoms persisted with crusting and ulceration from the nasal ala to the tip. A histopathological review of the recurrent nasal-tip lesions revealed that the 2014 biopsy showed superficial and focally infiltrative BCC with actinic keratosis, although only actinic keratosis was initially reported.

**Figure 1:**
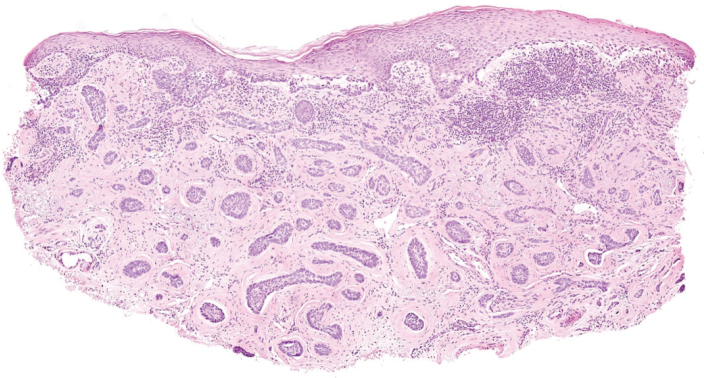
Histopathology from a punch biopsy of the left nasal region from August 2023 showing relapsed superficial and infiltrating basal cell carcinoma involving the margins, jagged and micronodular nests, and perineural growth.

**Figure 2:**
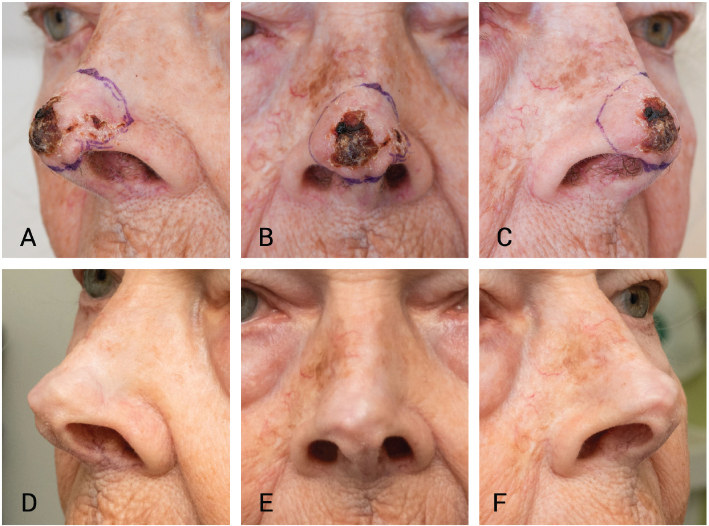
(A–C) Basal cell carcinoma on the nose at the time of planning contact brachytherapy, just before initiating the eribulin therapy. (D–F) Visit prior to the initiation of contact brachytherapy during a scheduled interruption of the eribulin treatment. After six treatment cycles, the basal cell carcinoma healed.

In August 2023, a biopsy of the left nasal ala revealed an infiltrative BCC (Figure 1). Given its central facial location, size, and growth pattern, the patient was referred for MMS. However, due to other health problems such as cholecystitis, followed by the diagnosis and treatment of abdominal leiomyosarcoma, this intervention was postponed. In February 2025, the BCC on the nose measured approximately 20 mm and appeared bulky and indurated (Figure 2A–C). Surgery for BCC was deemed inappropriate given the patient’s comorbidities and systemic disease burden. Additionally, resection is likely to require complex, multistage nasal reconstruction. A multidisciplinary tumour board recommended contact brachytherapy for BCC after completion or a pause in the oncological treatment of the recently diagnosed leiomyosarcoma.

### Metastatic leiomyosarcoma

In 2024, a liver lesion was detected on follow-up after a cholecystectomy for cholecystitis. Biopsy confirmed abdominal leiomyosarcoma. The tumour was inoperable, with a lesion occupying the right liver lobe and satellite lesions, with metastases limited to the right lobe. Enlarged lymph nodes and lung lesions were observed in the hepatoduodenal ligament of the patient. Investigations revealed the presence of skeletal metastases.

She initially received pazopanib, which was discontinued due to liver toxicity, followed by liposomal doxorubicin, which failed to control disease progression. In February 2025, systemic treatment was switched to eribulin mesylate for metastatic leiomyosarcoma (70% reduced dose). After six cycles, imaging performed at the end of July 2025 revealed a stable disease. Eribulin treatment was tolerated with only milder (grade 1–2) side effects (fatigue, diarrhoea, non-febrile neutropenia, and elevated liver enzymes) that were treated and followed up in outpatient care. The patient remained active and walked daily during the treatment period. Owing to the milder side effects, treatment was paused during the summer.

A new dermatological evaluation was performed in July 2025, during a pause in eribulin therapy in preparation for brachytherapy of the nasal tumour. Unexpectedly, the previously longstanding and infiltrative BCC completely disappeared both clinically and dermoscopically. The nasal tip appeared smooth and soft with slightly atrophic skin, freely mobile skin, and no signs of residual tumour (Figure 2D–F). Biopsy was not performed because of unequivocal clinical healing. At the follow-up visit for restarting eribulin 4 months later, the nose showed no signs of recurrence.

Next-generation sequencing (NGS) (Oncomine Childhood Cancer Assay) was performed on the leiomyosarcoma and showed an amplification of MDM2 (CNV253). MDM2 amplification is commonly seen in dedifferentiated liposarcomas but has also been described in leiomyosarcomas.

## Discussion

Eribulin is an antineoplastic drug that inhibits microtubules and is used to treat advanced breast cancer and soft-tissue sarcoma [5]. Eribulin is generally well tolerated, with mild side effects, and is occasionally used in later lines of therapy in patients with soft tissue sarcoma. It binds to the colchicine-binding site of tubulin [6, 7], inhibiting microtubule polymerisation and leading to mitotic arrest and cell death. Beyond its direct antiproliferative effect, eribulin remodels the tumour microenvironment by improving vascular perfusion and reversing epithelial-mesenchymal transition (EMT), which can enhance immune-mediated tumour control and reduce invasiveness. Microtubules are essential for various cellular processes, including mitosis, intracellular transport, maintenance of cell shape, and migration. In BCC, which is typically locally invasive rather than metastatic, disruption of microtubule function could potentially impair both proliferative and infiltrative properties and alter the interactions between tumour cells and the surrounding stroma or immune cells.

Given that canonical Hedgehog signalling in BCC depends on intact primary cilia for glioma-associated oncogene homologe 1 (GLI1) activation, eribulin may theoretically suppress GLI1-mediated transcription by disrupting microtubule dynamics required for ciliogenesis [11]. This could contribute to antitumour effects beyond mitotic inhibition.

Primary cilia, which are microtubule-based organelles essential for canonical hedgehog pathway activation, depend on intact microtubule dynamics for their structure and function [6, 7]. In BCC, primary cilia are required for GLI1 activation and hedgehog-dependent tumourigenesis [6]. Studies have demonstrated that INTU (Inturned), a ciliogenic protein, is aberrantly elevated in BCC and is essential for primary cilia formation and subsequent hedgehog signalling activation [11].

Eribulin’s disruption of microtubule dynamics could theoretically impair ciliogenesis or ciliary function, thereby suppressing GLI1-mediated transcription independent of smooothened inhibition. This mechanism is distinct from current HHIs and might explain the efficacy of eribulin in infiltrative BCC. However, it is important to observe that the relationship between microtubule inhibitors and primary cilia function is complex, as studies on other cancer types have shown variable effects on ciliary signalling [9]. Cytostatic treatments preceding eribulin may modulate the tumour microenvironment, facilitating a subsequent response to eribulin. In this case, complete regression of histologically confirmed BCC occurred during systemic eribulin treatment. The healing observed during eribulin therapy suggests a potential direct or indirect antitumour effect.

Tirbanibulin, a topical tubulin inhibitor approved for actinic keratoses [12], has also been tested on BCC with some reported activity, and clinical trials are planned, primarily for superficial BCC [10]. However, its efficacy may be limited by its shallow skin penetration. The effectiveness of systemically administered microtubule-targeting agents, such as eribulin, against deeper tumours, such as infiltrative BCC, requires further investigation. To the best of our knowledge, this is the first report on the efficacy of eribulin in BCC treatment. Although this is a single case, it raises important questions regarding the broader biological relevance of targeting microtubule function in BCC treatment.

## Conclusion

This case illustrates the complete regression of biopsy-confirmed BCC during systemic eribulin treatment for metastatic leiomyosarcoma. This finding was unexpected and occurred without the need for local therapies. This response supports the hypothesis that microtubule-targeting agents affect the biology of BCC. Eribulin’s systemic administration and mechanism of action involving microtubule inhibition and tumour microenvironment remodelling may offer a novel approach to managing difficult-to-treat BCCs.

The specific clinical circumstances and sequence of events made this observation possible, providing an unanticipated window into the potential antitumour effects of eribulin beyond its known indications. The favourable safety profile in the patient, with only grade 1–2 toxicities, compares favourably to the significant side effects associated with hedgehog inhibitors, which frequently lead to treatment discontinuation [4].

Further research is necessary to explore this potential therapeutic pathway, and increased attention to and documentation of concurrent BCC in patients receiving eribulin for sarcoma or other conditions may help to determine whether this effect can be consistently reproduced.

## Data Availability

The data supporting the findings of this case report consist of clinical facial photographs and histopathological images that contain potentially identifiable patient information. Due to ethical and legal restrictions, these data are not publicly available. Further information may be obtained from the corresponding author upon reasonable request, subject to appropriate approvals.
